# Energy-efficient traffic sign recognition using directly trained spiking neural networks and population decoding

**DOI:** 10.3389/fnins.2026.1771436

**Published:** 2026-05-15

**Authors:** Jonas V. Schulte, Steven Peters

**Affiliations:** Institute of Automotive Engineering, Technical University (TU) of Darmstadt, Darmstadt, Germany

**Keywords:** automated driving, energy-efficient perception, neuromorphic computing, population decoding, spiking neural networks, traffic sign recognition

## Abstract

Recognizing traffic signs is a fundamental perception task for automated driving systems and requires high accuracy under strict latency and energy constraints. Convolutional neural networks (CNNs) achieve strong performance but can be computationally demanding for embedded platforms. Spiking convolutional neural networks (SCNNs) offer an event-driven alternative that can reduce computation through sparse activity, yet their accuracy often degrades under very low-latency settings with few time steps. To improve spike-based inference under strict runtime constraints, we integrate a neural population decoding layer at the output stage and evaluate directly trained SCNNs with and without population decoding against a CNN baseline on the German Traffic Sign Recognition Benchmark (GTSRB). The best SCNN without population decoding achieved 98.85% test accuracy at 30 time steps, exceeding the CNN baseline of 98.38%. Population decoding improved performance in the low-latency regime, reaching 98.31% accuracy at a single time step, corresponding to an improvement of 0.56% over the SCNN without population decoding at the same temporal setting. Using an operation-based energy estimation, the SCNNs achieved over 14 times higher energy efficiency than the CNN at one time step. Overall, the results demonstrate that directly trained SCNNs can surpass a comparable CNN while enabling flexible trade-offs between accuracy, inference time, and energy efficiency. In particular, population decoding proves beneficial when operating under strict latency constraints.

## 1 Introduction

Traffic sign recognition is an important component in the development of advanced driver assistance systems (ADAS) and automated driving (AD) (Yaqoob et al., 2019). It enables vehicles to automatically detect and interpret traffic signs such as speed limits, stop signs, and right-of-way regulations. By reducing the risk of missing important traffic signs, such systems contribute to improved road safety and support reliable compliance with traffic regulations in automated driving systems.

Over the past decade, the development of reliable traffic sign recognition models has predominantly relied on conventional deep learning approaches, particularly convolutional neural networks (CNNs) (Triki et al., 2024). CNNs have demonstrated impressive accuracy across a wide range of vision tasks, including traffic sign recognition, by learning hierarchical feature representations directly from image data (LeCun et al., 2015). Their powerful feature extraction capabilities allow CNNs to distinguish complex patterns, such as those found in various traffic sign designs and styles, making them highly suitable for accurate image recognition tasks.

While CNNs excel in terms of accuracy, their energy demand and computational requirements present significant challenges when implementing these models in resource-constrained systems, particularly as the complexity of perception tasks continues to increase. These limitations arise from the dense, layer-by-layer computations required in CNNs (Howard et al., 2017), which can lead to significant energy consumption, especially in high-frequency tasks such as continuous visual perception in autonomous vehicles. This issue is further compounded by the need for embedded systems in vehicles to operate under strict energy budgets. Therefore, an effective alternative must be able to retain high accuracy while being more energy-efficient and capable of low-latency inference.

Neuromorphic computing has emerged as a promising approach to address these challenges by drawing inspiration from the structure and functionality of biological neural systems (Maass, 1997). Neuromorphic models, particularly spiking neural networks (SNNs), mimic the asynchronous and event-driven behavior of biological neurons, where information is transmitted only when specific conditions are satisfied. This event-driven paradigm allows SNNs to operate with sparse activity patterns, which can lead to potential energy savings compared to conventional CNNs that rely on continuous signal propagation (Pfeiffer and Pfeil, 2018).

However, SNNs face unique challenges due to their reliance on time-based spiking mechanisms, which introduce complex temporal dynamics. In real-time applications such as traffic sign recognition, these dynamics must unfold within a limited amount of time, making it difficult to achieve both high accuracy and low latency. As a result, SNNs often struggle to match the performance levels achieved by CNNs, particularly when efficiently processing spatial information under strict time constraints (Kim et al., 2023). Overcoming this limitation requires strategies that improve temporal efficiency without compromising predictive performance.

While traffic sign recognition is a well-established component of modern driver assistance and automated driving systems, its relevance extends beyond its immediate functionality. In the context of developing perception models for fully automated driving, traffic sign recognition provides a controlled yet challenging testbed that captures key aspects of more complex vision tasks. By focusing on object recognition, we aim to investigate core challenges in neuromorphic perception under real-world constraints and provide insights that are transferable to more demanding tasks such as general object detection and semantic scene understanding required for automated driving.

In this study, we investigate the potential of SNNs for perception tasks in automated driving using traffic sign recognition as a representative example. We address the challenge of balancing accuracy, energy efficiency, and computational complexity in spiking neural networks by exploring different model configurations, including varying numbers of time steps and the use of population decoding at the output layer.

Population decoding is a neuroscience-inspired technique in which groups of neurons collectively represent specific information, enhancing the robustness of neural representations (Averbeck et al., 2006). By exploiting population decoding, we aim to reduce the reliance on extensive temporal dynamics, enabling the network to achieve reliable predictions with fewer time steps. This approach distributes class information across multiple neurons rather than relying on spikes from a single output neuron.

Throughout this work, ANN and SNN refer to general model classes, while CNN and SCNN (spiking convolutional neural networks) denote the specific architectures used in this study. The main contributions of this work are summarized as follows:

1. We demonstrate that directly trained SCNNs can achieve competitive and, for selected temporal configurations, higher classification accuracy than a comparable CNN baseline on the German Traffic Sign Recognition Benchmark (GTSRB). All results are reported as mean and standard deviation over five independent training runs.
2. We investigate population decoding at the output layer as a strategy to improve classification performance under strict low-latency conditions. The results show that population decoding provides a consistent advantage when only a very small number of time steps is available.
3. We systematically quantify the energy efficiency of SCNNs compared to a CNN baseline by analysing activation sparsity and operation-based energy demand. Our results indicate that SCNNs can achieve up to 14.95 times higher energy efficiency in the single-time-step setting.
4. We analyse the trade-off between classification accuracy, number of time steps, and estimated energy demand across different SCNN configurations, providing practical guidance for selecting suitable operating points under varying application constraints.

Our results indicate that, given sufficient temporal integration, SCNNs can match or exceed the accuracy of a comparable CNN baseline while offering improved energy efficiency. These findings highlight the potential of SCNNs for real-time perception tasks in resource-constrained environments. The code for this work, including the trained model weights and the energy estimation tool, is publicly available at: https://github.com/TUDa-FZD/Spiking-Traffic-Sign-Recognition.

## 2 Background

In this section, we introduce the basic principles of SNNs and outline their advantages for energy-efficient computing. We explain how SNNs differ from conventional ANNs in terms of information processing and describe the spiking neuron model used in our architecture.

**Figure 1:**
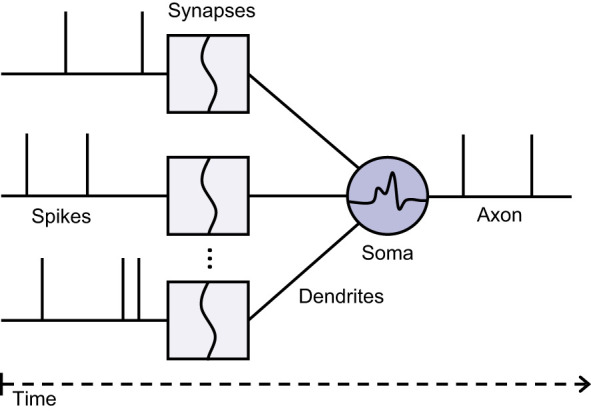
Schematic illustration of a spiking neuron receiving multiple synaptic inputs. Incoming spikes are weighted and accumulated at the soma, and an output spike is generated when the membrane potential exceeds a threshold.

SNNs are neural networks inspired by the behavior of biological neurons. Unlike conventional neural networks, which rely on continuous activations, SNNs communicate through discrete events called spikes. This event-driven paradigm allows neurons to remain inactive until their membrane potential reaches a threshold, resulting in sparse computations that can improve energy efficiency. A schematic illustration of a spiking neuron is shown in Figure 1.

**Figure 2:**
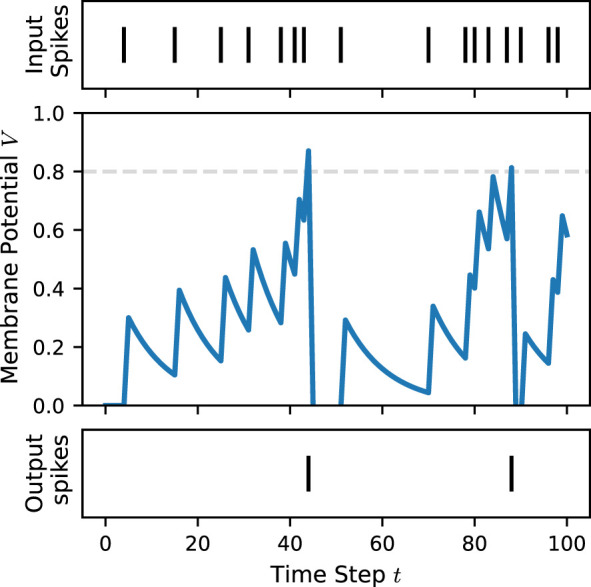
Example membrane potential dynamics of a leaky integrate-and-fire (LIF) neuron in response to multiple input spikes, simulated over 100 time steps using snnTorch (Eshraghian et al., 2023). The membrane potential increases with incoming spikes and generates an output spike once the firing threshold is reached.

One of the most commonly used models for describing spiking neuron dynamics is the leaky integrate-and-fire (LIF) model (Gerstner and Kistler, 2002), originally derived from Lapicque's early work (Brunel and Van Rossum, 2007). In this model, neurons integrate incoming signals over time, which modify the membrane potential. When the membrane potential exceeds a threshold, the neuron emits a spike and resets its state. In addition, a leakage term causes the membrane potential to decay over time in the absence of input. The LIF model is defined by the differential equation in Equation 1:


$$\begin{array}{l} \tau \frac{dV\left(t\right)}{dt}=-V\left(t\right)+RI\left(t\right) \end{array}$$ (1)

where *V*(*t*) represents the membrane potential at time *t*, τ is the membrane time constant (τ = *RC*), *R* is the membrane resistance, *C* is the membrane capacitance, and *I*(*t*) represents the input current. When *V*(*t*) reaches the threshold potential *V*_thr_, the neuron generates a spike according to Equation 2:


$$S(t)=\{\begin{array}{ll} 1, & \text{if }V(t)>V_{\text{thr}}\\ 0, & \text{otherwise} \end{array}$$ (2)

where *S*(*t*) denotes the spike at time *t*. Figure 2 illustrates the membrane potential dynamics of a LIF neuron simulated over multiple time steps.

Although the LIF model is a simplification and does not capture all biological neuron properties, such as adaptation or complex dendritic processing (Izhikevich, 2004), its simplicity makes it computationally efficient and well suited for large-scale SNN simulations. Because neurons only emit spikes when sufficiently stimulated, SNNs can exploit sparse activity patterns. This property makes them particularly attractive for energy-efficient computation on neuromorphic hardware.

## 3 Related work

Traffic sign recognition has undergone significant advancements, transitioning from traditional computer vision techniques to deep learning methods. Early approaches predominantly relied on manual feature extraction, utilizing techniques such as color-based segmentation, edge detection, and shape analysis to identify and classify traffic signs (Bahlmann et al., 2005; Maldonado-Bascón et al., 2007). While these methods were innovative for their time, they often suffered from sensitivity to variations in lighting conditions, occlusions, and complex backgrounds. This sensitivity led to limited robustness and accuracy in real-world scenarios. Moreover, the necessity for extensive tuning and hand-crafted features hindered their scalability and adaptability to new datasets or varying environmental conditions.

The emergence of ANNs, particularly CNNs, marked a pivotal shift in computer vision. CNNs excel at automatically extracting hierarchical features from images, capturing complex spatial patterns and variations inherent in traffic sign shapes and colors (Krizhevsky et al., 2012). Numerous studies have demonstrated the efficacy of CNNs in achieving state-of-the-art accuracy on large-scale datasets. For example, CNN-based models trained on the German Traffic Sign Recognition Benchmark (GTSRB) have achieved high accuracy, outperforming traditional methods (Ciregan et al., 2012). These results highlight the potential of CNNs for high-precision recognition tasks. However, the computational cost and energy demands associated with CNNs present challenges for real-time deployment in resource-constrained environments, such as autonomous vehicles.

Recent studies have applied SNNs to tasks such as image classification, gesture recognition, and speech processing, yielding promising results (Lee et al., 2016; Diehl and Cook, 2015). In addition, SNNs offer great potential for energy-efficient information processing. For instance, SNN-based architectures implemented on neuromorphic hardware have significantly reduced energy usage compared to conventional CNNs (Akopyan et al., 2015; Davies et al., 2018). Despite these advances, SNNs often struggle to match the accuracy of CNNs, especially in tasks with high variability such as traffic sign recognition. Researchers have proposed various methods, such as synaptic plasticity and hybrid SNN-ANN models, to bridge this performance gap, but these approaches often introduce additional complexity without fully achieving the desired balance of efficiency and accuracy (Wu et al., 2018).

To address these challenges, Xie et al. (2022) proposed a federated learning framework incorporating SNNs for privacy-preserving traffic sign recognition in vehicular networks. Their approach introduced neuronal receptive field encoding (NRFE), which combines spatial and pixel-level information for improved robustness against blurred or noisy traffic signs. While the framework achieved notable gains in accuracy and energy efficiency, it faces several challenges. The dependency on federated aggregation protocols introduces communication overhead and complicates participation consistency across devices. Moreover, the framework struggles with highly non-IID (non-independent and identically distributed) data distributions, which are prevalent in real-world vehicular networks. Additionally, the encoding complexity of the NRFE technique may increase computational demands, potentially hindering its feasibility for real-time applications.

Przewlocka-Rus and Kryjak (2022)) extended the application of SNNs by developing a convolutional spiking neural network (SCNN) for traffic sign classification using the leaky integrate-and-fire neuron model. The authors achieved up to 97% accuracy on the GTSRB dataset, demonstrating the effectiveness of spiking networks in this domain. However, achieving this accuracy required 100 time steps, leading to longer inference times compared to traditional CNNs. Despite the notable performance, the model exhibited an accuracy gap of 2.71% compared to the best-performing CNN reported by Arcos-García et al., which achieved an accuracy of 99.71% on the GTSRB (Arcos-Garćıa et al., 2018). This highlights the need for a sufficient trade-off between energy efficiency and real-time feasibility in SNNs.

Chen et al. (2024) proposed a hybrid CNN-SNN architecture deployed on FPGA platforms for traffic sign recognition. By incorporating a spatial attention mechanism and optimized neuron models, the system achieved a recognition accuracy of 99.22% with low energy consumption of 1.423 W. While this approach demonstrated excellent hardware efficiency and performance, it does not fully exploit the potential advantages of fully spiking architectures. The reliance on hybrid architectures instead of fully spiking implementations diminishes the inherent energy-saving advantages of SNNs, leaving room for further innovation in leveraging neuromorphic paradigms.

Another approach to addressing the trade-off between classification accuracy and real-time inference is the use of population coding schemes, which represent information through the collective activity of groups of neurons. This strategy has been proposed to improve the robustness and accuracy of spike-based representations, potentially enabling reliable predictions with reduced temporal resolution and, consequently, lower energy consumption.

Studies such as those by Pan et al. (2019) have demonstrated the benefits of population latency and phase coding in temporal classification tasks, achieving near-perfect accuracy on audio datasets. However, it remains unclear whether these advantages transfer to image recognition problems. In addition, the computational overhead and scalability challenges associated with population decoding in larger networks have not yet been fully explored.

While previous studies have investigated spiking architectures and hybrid CNN–SNN approaches for traffic sign recognition, the potential of directly trained fully spiking convolutional networks under strict latency constraints has not been systematically studied. In particular, the role of population decoding in improving low-latency inference performance for vision tasks remains insufficiently explored.

## 4 Methodology

In this section, we describe the architecture and training method of the SCNNs used in this work. We outline the key components of our model, including the input representation, the population decoding strategy, and the training procedure based on surrogate gradients. In addition, we describe the CNN baseline used for comparison.

### 4.1 SCNN architecture

**Figure 3:**
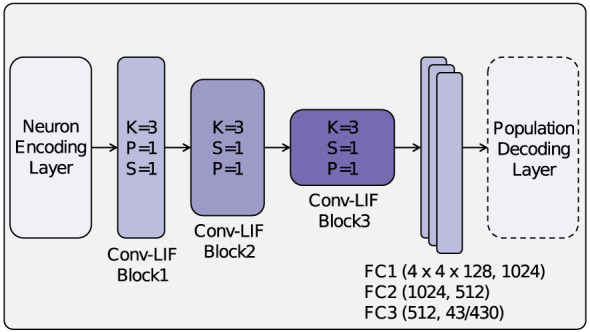
Architecture of the proposed SCNN. The model consists of a trainable image encoding layer, three convolutional LIF blocks with kernel size (K), stride (S), and padding (P), followed by three fully connected (FC) layers and an optional population decoding layer at the output.

**Figure 4:**
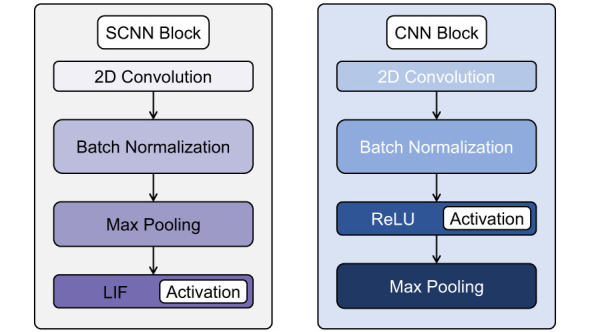
Comparison of the convolutional blocks used in the SCNN and the CNN backbone. In the SCNN, max pooling is applied before the spiking activation, whereas in the CNN the activation precedes the pooling operation.

Our main model for the traffic sign recognition task is a spiking convolutional neural network (SCNN), which combines the hierarchical feature extraction of CNNs with the temporal dynamics of spiking neurons. A detailed illustration of the network architecture, including its convolutional and fully connected layers, is provided in Figures 3, 4. The SCNN consists of three convolutional layers, each followed by batch normalization, max pooling, and LIF neurons, which introduce spiking dynamics by integrating incoming signals and generating spikes over time. To systematically analyse the effect of population decoding, we train two variants: one with population decoding in the output layer, where the class representation is distributed across multiple neurons, and another with a conventional single-neuron-per-class representation, where each class is represented by a single neuron.

The architecture is structured as follows:

- *Convolutional Layers*: The SCNN consists of three convolutional layers with 32, 64, and 128 filters in the first, second, and third layers, respectively. Each layer applies a 3 × 3 kernel with a padding of 1 to preserve spatial dimensions. Batch normalization is applied after each convolution to stabilize training and improve convergence. Max pooling is applied before the spiking activation to ensure that the maximum values are selected from continuous activations before they are converted into binary spikes. A kernel size of 2 and a stride of 2 are used to progressively reduce spatial resolution. The different arrangement of layers in the SCNN backbone compared to the CNN baseline is illustrated in Figure 4.
- *Leaky Integrate-and-Fire (LIF) Neurons*: After max pooling, LIF neurons introduce spiking dynamics by integrating the input current over time and generating spikes based on their membrane potential. The leakage parameter (β = 0.9) controls the decay rate, ensuring that neurons retain a short-term memory of previous activations while remaining responsive to new inputs.
- *Fully Connected Layers*: After feature extraction, the output is flattened and passed through three fully connected layers. The first two layers contain 1024 and 512 neurons, respectively, and use LIF neurons to maintain temporal dynamics throughout the network. The output layer contains either 43 or 430 neurons, depending on whether population decoding is used.

During both training and inference, the SCNN processes input data over a fixed number of time steps *t*, allowing spikes to propagate through the network and accumulate in the output layer. By exploiting both spatial and temporal information, the network aims to balance classification accuracy and computational efficiency. A higher number of time steps enables more precise temporal integration, which can improve classification accuracy. However, it also increases computational cost due to the larger number of spike-based operations. To evaluate this trade-off, models with different time-step configurations are compared, and the impact on accuracy and computational efficiency is analyzed.

### 4.2 Input representation

The representation of input data plays a critical role in the performance of SNNs. Unlike conventional artificial neural networks (ANNs), which process continuous-valued activations, SNNs operate on discrete spike events distributed over time. Therefore, an input encoding mechanism is required to transform static image data into a representation suitable for spike-based computation.

Several coding strategies have been proposed (Auge et al., 2021). Rate coding represents information through the firing frequency of neurons, while temporal and latency coding encode pixel intensities in the precise timing of spikes. In contrast, trainable coding allows the network to learn an appropriate input representation directly from the data rather than relying on a predefined encoding scheme.

In this work, we adopt a trainable coding approach in which the first convolutional layer directly processes the raw input image to extract low-level feature maps. These feature maps are replicated over *t* time steps, allowing the convolutional layer to act as a trainable encoder that optimizes the input representation during training. The resulting activations can be interpreted as input currents to the LIF neurons, which subsequently generate spikes according to their membrane dynamics. This approach avoids manually designed encoding schemes while providing an efficient transformation of visual input for SNN processing.

### 4.3 Population decoding layer

In conventional SNN classification architectures, each output neuron corresponds to a single class, and the final prediction is determined by selecting the neuron with the highest spike count. While this approach is simple, it can be sensitive to noise and may require multiple time steps to produce reliable activations.

To improve spike-based inference under strict latency constraints, we incorporate population decoding, a biologically inspired strategy in which information is represented by the collective activity of multiple neurons per class (Averbeck et al., 2006). By distributing class information across a population of neurons rather than relying on a single output unit, the representational capacity of the output layer is increased. This can enable more reliable classification with fewer time steps, which is particularly beneficial for low-latency and energy-efficient inference.

Instead of mapping each class to a single neuron, each class is represented by a population of 10 neurons, resulting in a total of 430 output neurons. The final prediction is obtained by summing the spike counts of the neurons associated with each class and selecting the class with the highest cumulative spike count.

While population decoding has been suggested to improve robustness by distributing information across multiple neurons and potentially enabling earlier classification, its effectiveness depends on the interaction between population size and temporal dynamics. In this work, we therefore evaluate its impact across different numbers of time steps to determine whether it provides advantages over conventional single-neuron output representations.

### 4.4 Surrogate gradient learning

Training deep SNNs presents unique challenges due to the non-differentiability of spike activations. Unlike ANNs, where gradient-based optimization can be applied directly, SNNs rely on discrete spike events, making standard backpropagation inapplicable.

One approach to obtain a trained SNN is ANN-to-SNN conversion, where a pre-trained ANN is mapped onto an SNN by replacing activation functions with spiking neurons and normalizing weight magnitudes to account for rate-coded spike activity (Diehl et al., 2015; Rueckauer et al., 2017; Sengupta et al., 2019). While this approach can achieve high accuracy, it typically requires a large number of time steps due to the reliance on rate coding and does not fully exploit the temporal processing capabilities of SNNs (Pfeiffer and Pfeil, 2018).

To leverage the event-driven nature of SNNs, the models in this work were trained directly using backpropagation through time (BPTT) (Werbos, 1990). By formulating SNNs as recurrent neural networks (RNNs), synaptic weights can be optimized across multiple time steps. However, direct training is complicated by the Heaviside step function *S*(*t*) that defines spike generation. Since *S*(*t*) has a derivative of zero almost everywhere and is undefined at the threshold, standard gradient-based optimization cannot be applied.

To address this issue, surrogate gradient learning (Neftci et al., 2019) was used. The derivative of *S*(*t*) is replaced by a continuous approximation σ(*t*), enabling gradient propagation during training. In this work, the surrogate function and its derivative, given in Equations 3 and 4, were used, where V(t) denotes the membrane potential of the spiking neuron:


$$\begin{array}{l} \sigma \left(t\right)=\frac{1}{\pi }\arctan\left(\pi \frac{\alpha }{2}V\left(t\right)\right) \end{array}$$ (3)

$$\begin{array}{l} \frac{\partial \sigma \left(t\right)}{\partial V\left(t\right)}=\frac{1}{\pi }\frac{1}{1+{\left(\pi \frac{\alpha }{2}V\left(t\right)\right)}^{2}} \end{array}$$ (4)

Using this surrogate function ensures stable gradient flow during training and enables the direct optimization of multi-layer SNNs. Compared to ANN-to-SNN conversion, direct training allows the network to learn temporal dynamics directly and optimize spike-based representations for event-driven computation.

### 4.5 CNN baseline model

To provide a reference for performance evaluation, a CNN baseline model was implemented for traffic sign classification. The architecture mirrors the backbone of the SCNN but replaces spiking neurons with conventional Rectified Linear Unit (ReLU) activations. The model consists of three convolutional layers, each followed by batch normalization, ReLU activation, and max pooling, and three fully connected layers for classification.

The architecture includes the following components:

- *Convolutional Layers*: The CNN contains three convolutional layers with 32, 64, and 128 filters, respectively. Each layer uses a 3 × 3 kernel with padding 1 to preserve spatial dimensions. Batch normalization stabilizes training, while ReLU activation introduces nonlinearity. Max pooling with kernel size 2 and stride 2 progressively reduces spatial resolution.
- *Fully Connected Layers*: After feature extraction, the feature maps are flattened and passed through three fully connected layers. The first two layers contain 1024 and 512 neurons with ReLU activations. The final layer consists of 43 neurons, corresponding to the number of traffic sign classes, and outputs the logits for classification.

This CNN baseline provides a direct comparison to the SCNN architecture, enabling the evaluation of differences in accuracy, computational efficiency, and energy consumption.

## 5 Experiments and results

This section presents the experimental setup and results on the GTSRB dataset. We evaluate the classification accuracy and energy efficiency of the models across different time-step configurations and analyse the impact of population decoding. The results are also compared with the CNN baseline.

### 5.1 Dataset and experimental setup

For this study, the German Traffic Sign Recognition Benchmark (GTSRB) (Stallkamp et al., 2012) was used. The dataset contains 51,839 images divided into 43 classes, with 39,209 samples in the original training set and 12,630 samples in the official test set. The images exhibit significant variations in lighting conditions, viewing angles, distances, and backgrounds, making GTSRB a widely used benchmark for traffic sign recognition.

To obtain a validation set for model selection, a stratified 10% split of the original training set was used, preserving the class distribution. The resulting validation set was used exclusively for early stopping and learning-rate scheduling, while the official test set was reserved strictly for final performance evaluation.

**Figure 5:**
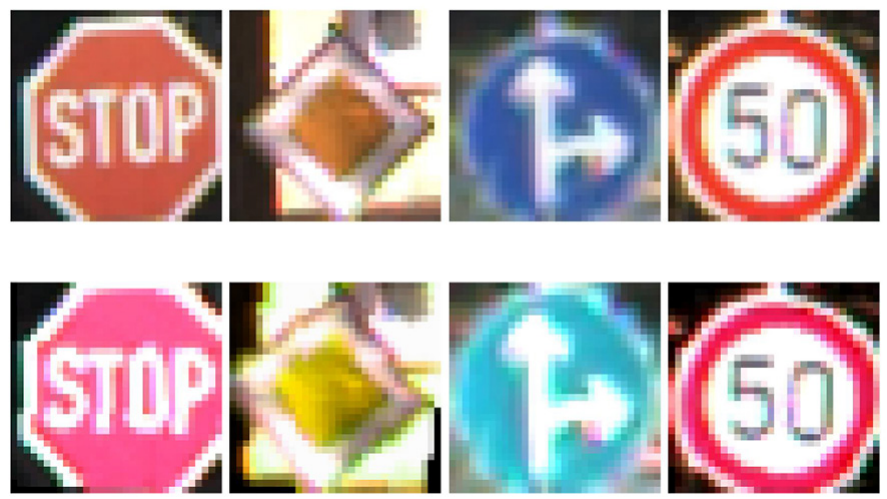
Sample images of the GTSRB (Stallkamp et al., 2012) training dataset with **(Bottom)** and without data augmentation **(Top)**. The images are reproduced/adapted from the dataset for illustrative purposes. The Zenodo record for GTSRB lists the dataset under Creative Commons Attribution 4.0 International (CC BY 4.0). Source: German Traffic Sign Recognition Benchmark (GTSRB), Real-Time Computer Vision Group / Ruhr University Bochum.

To ensure compatibility with both CNN and SCNN models, all images were resized to 32 × 32 pixels and normalized to zero mean and unit variance. Data augmentation was applied during training using random rotations, resized cropping, and color jittering, as illustrated in Figure 5. During validation and testing, only resizing and normalization were applied.

All models were trained using PyTorch and snnTorch (Eshraghian et al., 2023) on an NVIDIA RTX 4090 GPU. Synaptic weights were optimized using the Adam optimizer with an initial learning rate of 1 × 10^−3^. The learning rate was adaptively reduced using a ReduceLROnPlateau scheduler, which decreases the learning rate by a factor of 0.5 when the validation loss does not improve for five consecutive epochs, with a minimum learning rate of 1 × 10^−6^. The batch size was set to 128, and early stopping with a patience of 10 epochs was applied.

Different loss functions were used depending on the model architecture. The CNN baseline was trained using the standard cross-entropy loss commonly used for CNN classification models. In contrast, the SCNN models were trained using mean squared error (MSE) applied to the accumulated spike counts over time.

In preliminary experiments, we compared MSE and cross-entropy loss for training the SCNN models across multiple time-step configurations. The comparison on the validation set showed that MSE generally resulted in more stable optimization and slightly higher validation accuracy, particularly for very small numbers of time steps. This behavior can be explained by the fact that the SCNN output represents accumulated spike counts rather than probability logits, making regression-based objectives such as MSE better aligned with the spike-based representation.

During each training iteration, the neuron states of the SCNNs were reset at the beginning of each forward pass to avoid dependencies between consecutive samples.

The performance of the SCNNs was evaluated across multiple time steps (1, 3, 5, 10, 20, and 30). In addition, SCNNs were trained both with and without population decoding, where classification decisions in the population-coded variant were obtained by summing the spike counts across the neurons assigned to each class.

To provide a comprehensive evaluation, classification accuracy, computational complexity, and energy efficiency were measured. The accuracy of the CNN was determined using the highest softmax probability, while the accuracy of the SCNN was determined by selecting the class with the highest cumulative spike count. FLOPs were computed for each model and combined with activation sparsity to estimate relative energy consumption.

### 5.2 Analysis on accuracy and time steps

The SCNN models were evaluated over several time steps (1, 3, 5, 10, 20, and 30) to analyse the relationship between temporal integration and classification accuracy. As shown in Table 1, the SCNN without population decoding achieved 97.75% (±0.20) at a single time step. Its accuracy increased with the number of time steps, reaching 98.85% (±0.18) at 30 time steps. This trend indicates that the model benefits from additional temporal integration, although the gains beyond 5–10 time steps become comparatively small.

**Table 1:** Accuracy (mean ± std in %) of the SCNN without and with population decoding (PD) and of the baseline CNN model on the test set of the GTSRB.

Accuracy [%]						
Time steps	1	3	5	10	20	30
SCNN	97.75 ± 0.20	98.52 ± 0.16	98.59 ± 0.18	98.76 ± 0.07	98.73 ± 0.14	**98.85** **±0.18**
SCNN + PD	**98.31** **±0.31**	98.56 ± 0.22	98.66 ± 0.18	98.55 ± 0.19	98.71 ± 0.21	98.51 ± 0.18
CNN	98.38 ± 0.28	–	–	–	–	–

Results are averaged over five independent runs with different random seeds. Configurations that are both more accurate and more energy-efficient (Table 3) than the CNN baseline are highlighted in green. Bold values indicate the best-performing result within the respective comparison.

Incorporating population decoding improved performance particularly in the low-latency regime. At a single time step, the SCNN with population decoding achieved 98.31% (±0.31), outperforming the SCNN without population decoding by 0.56%. At 3 and 5 time steps, the population-coded model remained slightly higher or comparable, with accuracies of 98.56% (±0.22) and 98.66% (±0.18), respectively. At three time steps, the population-coded model already exceeded the CNN baseline by 0.18% in terms of mean accuracy. For larger numbers of time steps, however, the advantage diminished, and the SCNN without population decoding achieved the highest overall mean accuracy.

The CNN baseline achieved 98.38% (±0.28) test accuracy. Compared with this baseline, the SCNN with population decoding nearly matched CNN performance already at a single time step and continued to outperform it for larger numbers of time steps. The SCNN without population decoding surpassed the CNN baseline from five time steps onward and achieved the highest overall performance at 30 time steps. Overall, these results show that directly trained SCNNs can provide a favorable trade-off between latency and accuracy, while population decoding is especially beneficial when only very few time steps are available.

To contextualize the performance of the proposed models, Table 2 compares our results with representative CNN, hybrid CNN–SNN, and fully spiking approaches reported in the literature for the GTSRB dataset. While several deep CNN architectures achieve higher absolute accuracy under different experimental conditions, the proposed SCNN models provide competitive accuracy while enabling flexible trade-offs between inference latency and energy efficiency.

**Table 2:** Comparison of traffic sign recognition approaches on the GTSRB dataset.

**Method**	**Model type**	**Accuracy [%]**	**Time steps**	**Notes**
Previous work				
Ciregan et al. (2012)	CNN	99.46	–	Multi CNN ensemble
Arcos-Garćıa et al. (2018)	CNN	99.71	–	CNN + spatial transformer
Chen et al. (2024)	Hybrid CNN-SNN	99.22	–	CNN-Attention + SCNN
Przewlocka-Rus and Kryjak (2022)	Fully spiking	97.0	100	ANN-SNN-Conversion
This work				
CNN	CNN	98.38 ± 0.28	–	Baseline
SCNN	Fully spiking	**98.85** **±0.18**	30	Best accuracy
SCNN + PD	Fully spiking	**98.31** **±0.31**	1	Single time step

The table summarizes representative CNN, hybrid CNN–SNN, and fully spiking methods reported in the literature together with the models evaluated in this work. Bold values indicate the best-performing result within the respective comparison.

### 5.3 Energy efficiency estimation

To estimate the energy consumption of SCNN models and compare them with the CNN baseline, we analyse the number of inference operations and the spike rate per layer. The estimation follows a commonly used analytical model that approximates energy consumption by scaling the number of operations with fixed energy costs (Kim et al., 2022; Barchid et al., 2023; Rathi and Roy, 2020). The operation count of SCNN layers is derived from the corresponding CNN formulation, since convolutional and fully connected layers share the same computational structure. Therefore, the number of operations in a conventional model represents a dense upper bound, which is scaled according to the observed spike activity to approximate the effective computation performed by the SCNN.

This operation-level approximation does not capture system-level effects such as memory accesses, data movement, or peripheral circuitry, which may influence the actual energy consumption of deployed systems. On neuromorphic hardware such as22 Intel Loihi (Intel Corporation, Santa Clara, CA, USA; Davies et al., 2018) or IBM TrueNorth (IBM Corporation, Armonk, NY, USA; Cheng et al., 2017), computations are executed in an event-driven manner with different memory access patterns than conventional processors. While the sparse spike activity of SCNNs can reduce the number of executed operations, hardware-specific factors such as irregular spike-driven memory access and communication overhead may limit the achievable efficiency gains in practice. Therefore, the reported energy efficiency values should be interpreted as architecture-independent indicators rather than direct hardware-level measurements.

The activation sparsity in SCNNs can be quantified using the spike rate RS per layer l, defined in Equation 5 as the average firing rate per neuron over the temporal window (Han et al., 2020; Yao et al., 2025):


$$\begin{array}{c} R_{S}\left(l\right)=\frac{n_{S}\left(l\right)}{n_{N}\left(l\right)\times T} \end{array}$$ (5)

where *n*_*S*_(*l*) denotes the total number of spikes emitted by layer *l* aggregated over all time steps, *n*_*N*_(*l*) represents the total number of neurons in that layer, and *T* is the number of time steps. Accordingly, *R*_*S*_(*l*) reflects the average spike activity per neuron and per time step over the full temporal window.

For the CNN, the total number of floating point operations (FLOPs) for convolutional and fully connected layers is given by:


$${\text{FLOPs}}_{\text{CNN}}(l)=\{\begin{array}{ll} k^{2}\times O^{2}\times C_{\text{in}}\times C_{\text{out}}, & \text{if }l=\text{Conv}\\ C_{\text{in}}\times C_{\text{out}}, & \text{if }l=\text{Linear} \end{array}$$ (6)

where *k* is the kernel size, *O* is the spatial size of the output feature maps, and *C*_in_ and *C*_out_ denote the number of input and output channels, respectively. Equation 6 captures the dominant weight-related operations in convolutional and fully connected layers. Bias additions are neglected, as their contribution to the overall operation count is negligible and does not affect the relative comparison between the CNN baseline and the SCNN models.

Based on this formulation, the computational cost of SCNN layers can be expressed by scaling the dense CNN operations according to the observed spike activity. Since the spike rate *R*_*S*_(*l*) represents the average firing rate per neuron and per time step, the factor *R*_*S*_(*l*) × *T* reflects the relative fraction of operations that are effectively executed compared to dense CNN computation. This captures the fact that SCNN computations only occur when spikes are generated, thereby reducing the effective number of operations per layer *l*, as calculated using Equation 7:


$$\begin{array}{c} {\text{FLOPs}}_{\text{SCNN}}\left(l\right)={\text{FLOPs}}_{\text{CNN}}\left(l\right)\times R_{S}\left(l\right)\times T \end{array}$$ (7)

This approximation assumes that the computational structure of SCNN layers is comparable to that of the corresponding CNN layers, while activation sparsity reduces the effective number of operations.

It should be noted that the population decoding configuration increases the number of neurons in the output layer from 43 to 430, resulting in an approximately tenfold increase in the computational cost of the final fully connected layer. However, this increase has only a minor impact on the overall computation, as the final layer contributes a small fraction of the total workload and the convolutional layers dominate the operation count. Consequently, the effect of population decoding on the total energy estimation remains limited, as reflected in the similar energy efficiency values reported in Table 3.

**Table 3:** Energy efficiency factors of the SCNN models relative to the CNN baseline on the GTSRB dataset.

Energy efficiency factor *E*_CNN_/*E*_SCNN_						
Time steps	1	3	5	10	20	30
SCNN	14.95	3.88	2.47	1.37	0.64	0.41
SCNN + PD	14.07	3.76	2.40	1.32	0.63	0.41

The values denote the ratio *E*_CNN_/*E*_SCNN_, where larger values indicate higher energy efficiency of the SCNN compared to the CNN. Configurations that are more energy-efficient than the CNN baseline are highlighted in green.

To estimate the total energy demand, we rely on the operation-level energy costs reported by Horowitz (2014). According to this, each multiply-accumulate (MAC) operation in a CNN consumes approximately *E*_MAC_ = 4.5 pJ, while an accumulate (AC) operation in a SCNN requires only *E*_AC_ = 0.9 pJ. This distinction is based on the fact that MAC operations involve both multiplication and accumulation, whereas AC operations in SNNs typically bypass the need for multiplications due to binary spike representations.

Thus, the total energy demand for the CNN is computed using Equation 8:


$$\begin{array}{c} E_{\text{CNN}}\approx \underset{l}{\sum }{\text{FLOPs}}_{\text{CNN}}\left(l\right)\times E_{\text{MAC}} \end{array}$$ (8)

For SCNNs, the corresponding energy demand is given by Equation 9:


$$\begin{array}{c} E_{\text{SCNN}}\approx \underset{l}{\sum }{\text{FLOPs}}_{\text{SCNN}}\left(l\right)\times E_{\text{AC}} \end{array}$$ (9)

**Figure 6:**
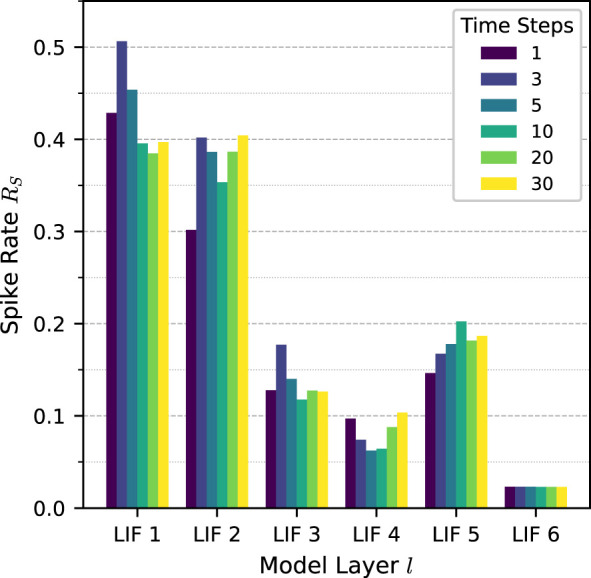
Spike rate per layer *R*_*S*_(*l*) of the SCNN with population decoding across different numbers of time steps. Early convolutional layers exhibit higher spike activity, while deeper layers show progressively lower spike rates, indicating increasing sparsity toward the output layer.

Figure 6 shows the spike rates per layer *R*_*S*_(*l*) for the SCNN with population decoding across different numbers of time steps. A clear trend can be observed across the network depth. The early convolutional layers exhibit comparatively high spike activity, whereas the deeper layers show significantly lower firing rates, indicating increasing sparsity toward the output layers.

Across different numbers of time steps, the spike rate per layer remains within a comparable range, reflecting the normalized definition in Equation 5. Consequently, the computational workload of SCNNs is determined by the product *R*_*S*_(*l*) × *T*. Increasing the number of time steps therefore leads to a higher number of operations, even if the spike rate itself changes only marginally.

Finally, the energy efficiency gain of SCNNs is quantified by the ratio *E*_CNN_/*E*_SCNN_, indicating how many times more energy-efficient the SCNN is compared to the CNN baseline. The results are summarized in Table 3. The largest efficiency gain is observed at a single time step, where the SCNN achieves up to 14.95 times higher estimated energy efficiency.

## 6 Discussion

Evaluating accuracy and energy efficiency across different numbers of time steps reveals a fundamental trade-off between temporal integration and computational efficiency in SCNNs. Increasing the number of time steps generally improves classification accuracy, as the network can accumulate more spike-based evidence. However, this also increases the number of spike-based operations and therefore reduces the relative energy-efficiency advantage of SCNNs compared to conventional CNNs.

At a single time step, both SCNN variants achieve an estimated energy efficiency that is more than 14 times higher than that of the CNN baseline. As the number of time steps increases, this advantage gradually diminishes. According to the operation-based estimate, the SCNN without population decoding is no longer more energy-efficient than the CNN at 30 time steps. This indicates that the energy benefits of spike-based computation depend strongly on the temporal operating point of the network.

Population decoding primarily improves classification performance in the low-latency regime but provides limited benefit at larger numbers of time steps. By distributing class information across multiple output neurons, population decoding increases the representational capacity of the output layer and stabilizes decisions when only a small number of spikes is available. As the number of time steps increases, however, temporal integration allows the network to accumulate sufficient spike evidence even with a single-neuron output representation. Consequently, the advantage of the larger output population diminishes, and the SCNN without population decoding can achieve slightly higher peak accuracy.

**Figure 7:**
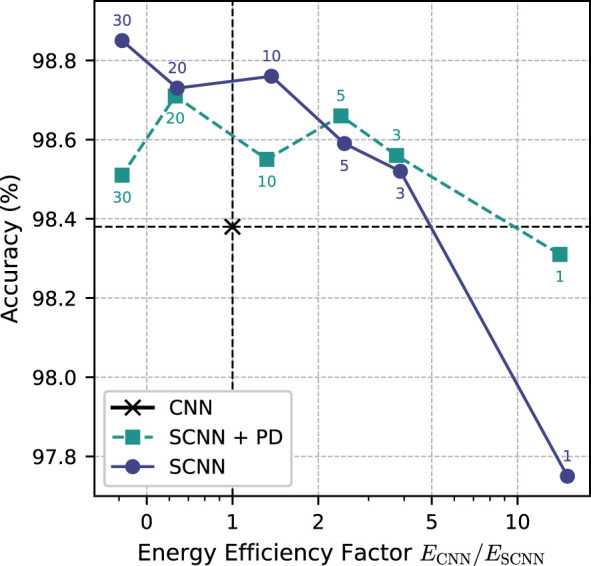
Trade-off between classification accuracy and estimated energy efficiency of SCNN models with and without population decoding (PD) compared to the CNN baseline. Each marker corresponds to a model evaluated with a specific number of time steps (annotated next to the points). The horizontal dashed line indicates the accuracy of the CNN baseline, while the vertical dashed line marks equal energy consumption relative to the CNN. Configurations in the upper-right quadrant therefore represent models that are both more accurate and more energy-efficient than the CNN baseline.

The trade-off between accuracy and energy efficiency is illustrated in Figure 7. In the low-latency regime, the SCNN with population decoding is particularly attractive, achieving 98.31% accuracy at a single time step while remaining 14.07 times more energy-efficient than the CNN baseline. Conversely, when maximum accuracy is the primary objective, the SCNN without population decoding is preferable, reaching 98.85% at 30 time steps, but with a reduced energy-efficiency advantage relative to the CNN. Particularly notable are the configurations located in the upper-right quadrant of Figure 7, as these models are both more accurate and more energy-efficient than the CNN baseline and therefore represent an attractive operating point.

Overall, these results suggest that population decoding is most beneficial in scenarios with strict latency constraints, where only a small number of time steps is available. For applications that allow longer temporal integration, the SCNN without population decoding can achieve the highest classification accuracy, while the relative energy-efficiency advantage compared to conventional CNNs becomes smaller.

While the presented results demonstrate promising trade-offs between accuracy, latency, and energy efficiency, several limitations of this study should be acknowledged that provide directions for future research. First, the evaluation was conducted exclusively on the GTSRB dataset. Although GTSRB is a well-established benchmark for traffic sign classification, it does not capture the full diversity of real-world perception scenarios. Evaluating the proposed approach on additional datasets will therefore be important to better understand its generalization across different domains and visual conditions.

Furthermore, the experiments focus on a single-object classification task. While traffic sign recognition represents an important perception component in automated driving systems, more complex perception tasks such as multi-object detection, scene understanding, or perception under challenging environmental conditions (e.g., adverse weather, occlusions, or nighttime scenarios) were not addressed in this study.

Applying the proposed SCNN architecture to higher input resolutions or more complex datasets introduces several scalability challenges. Increasing the input resolution enlarges the spatial dimensions of intermediate feature maps, leading to a proportional growth in the number of neurons and their internal states that must be maintained across time steps, thereby increasing the memory footprint.

Furthermore, more complex visual scenes are likely to activate a larger fraction of neurons simultaneously, which can lead to higher spike rates and reduced activation sparsity. Since the efficiency advantages of spiking neural networks rely on sparse activity, higher spike rates can diminish their potential energy benefits, as observed in prior work (Pfeiffer and Pfeil, 2018; Sorbaro et al., 2020). Overall, both input resolution and dataset complexity directly affect spike activity, memory requirements, and computational cost, highlighting the importance of evaluating scalability on more challenging datasets in future work.

Finally, the energy efficiency analysis in this study relies on an analytical operation-level model. While this approach provides an architecture-independent estimate that enables a consistent comparison between CNN and SCNN models, it does not capture the full system-level energy costs of real hardware implementations. Future work should therefore evaluate the proposed models on dedicated neuromorphic hardware platforms to obtain more realistic hardware-level energy measurements and to experimentally validate real-time latency on both GPU-based systems and neuromorphic hardware.

## 7 Conclusion

This study investigated the use of SCNNs for energy-efficient traffic sign recognition using the GTSRB dataset. A baseline CNN was compared with SCNN models across multiple time steps and decoding strategies to analyse the trade-off between classification accuracy and computational efficiency.

The results demonstrate that directly trained SCNNs can achieve competitive accuracy and, for certain temporal configurations, even higher mean accuracy than a comparable CNN baseline. At the same time, SCNNs provide substantial energy-efficiency advantages in low-latency settings due to sparse spike-based computation. In particular, population decoding proved beneficial when only a very small number of time steps was available, whereas the SCNN without population decoding achieved the highest overall mean accuracy at larger numbers of time steps.

Overall, the findings indicate that SCNNs provide a promising balance between energy efficiency and recognition accuracy, making them well suited for resource-constrained perception systems. Future work should validate the proposed models on neuromorphic hardware platforms to obtain hardware-level energy and latency measurements. In addition, adaptive time-stepping strategies and improved decoding mechanisms may further enhance the efficiency and scalability of spike-based perception models for more complex vision tasks in automated systems.

## Data Availability

The original contributions presented in the study are included in the article/supplementary material, further inquiries can be directed to the corresponding author.
